# 25 Hydroxyvitamin D and Cytokine Profile in Patients With Relapsing-Remitting Multiple Sclerosis

**DOI:** 10.7759/cureus.61534

**Published:** 2024-06-02

**Authors:** Georgi S Slavov, Mariya G Manova, Ivanka I Kostadinova

**Affiliations:** 1 Department of Neurology, Medical University of Plovdiv, Plovdiv, BGR; 2 Department of Pharmacology, Medical University of Plovdiv, Plovdiv, BGR

**Keywords:** immunoregulatory potential, deficit severity, cytokines, vitamin d, multiple sclerosis

## Abstract

In experimental allergic encephalomyelitis, the severity of the deficiency is associated with the loss of axons, and it is likely that cytotoxic T-cells 8 (CD8 T) play an important role. In relapsing-remitting multiple sclerosis, there is a correlation between the inflammatory activity in the lesion and the transection of axons. To understand the pathological mechanisms, it is important to evaluate the changes in serum concentrations of pro- and anti-inflammatory cytokines during the disease course. A total of 46 patients and 40 healthy individuals participated in an open-label, prospective, case-control study from 2012 to 2014. The serum concentrations of cytokines were measured using enzyme-linked immunosorbent assay (ELISA). An immune imbalance was observed during relapse and remission phases compared to the control group. During relapse, the levels of interferon-gamma (IFN-γ) were significantly higher compared to those in remission (p=0.017). During remission, there was an improvement in the deficiency (p<0.001), and the anti-inflammatory cytokines transforming growth factor-beta (TGF-β) and interleukin 4 (IL4) increased compared to those in relapse (p=0.006; p=0.009). A correlation was found between the serum concentrations of tumor necrosis factor-alpha (TNF-α) and Expanded Disability Status Scale (EDSS) during relapse (correlation coefficient: 0.301; significance (Sig.) (2-tailed 0.042). During the exacerbation, there was a moderate relationship between interleukin 17 (IL17) and 25-hydroxyvitamin D (25(OH)D) (P (p-value (probability value) = 0.02)). TNF-α, IFN-γ, IL17, and TGF-β serum levels are criteria for evaluating immune inflammatory activity during relapse and remission periods.

## Introduction

Multiple sclerosis (MS) is a neurodegenerative disease of the central nervous system (CNS) with an immune-related genesis and a complex etiology. A study in our country established a relapsing-remitting (RR) phenotype in 80% of the MS cohort [1]. The pattern of immune response to myelin antigens has not been definitively defined. Mediators of inflammation in certain stages protect neurons from degenerative disorders [2,3]. Experimental results show a relationship between changes in vitamin D concentrations, pattern development and severity of deficiency [4]. The immunoregulatory activity of vitamin D is determined by: presence of multiple target 1,25-dihydroxyvitamin D (1,25(OH)2D) genes in immune cells; the ability of immune cells (macrophages, dendritic, T and B lymphocytes) to convert 25-hydroxyvitamin D (25(OH)D) to the biologically active metabolite 1,25(OH)2D; production of 1,25(OH)2D by astrocytes and its participation in myelin production in the CNS [5]. Clinical observations present mixed results on the relationship between dynamics in serum levels of the vitamin, changes in immune indicators of disease activity, severity of deficiency, and gadolinium (Gd-enhanced lesions) [6-9]. In the Bulgarian population, the levels of 25(OH)D accepted for sufficiency (>50 nmol/l), insufficiency (25-29.99 nmol/l), and deficiency (<25 nmol/l) are identical to those indicated at the Consensus Conference in Germany (2012), which allows to comparatively analyze data from researches in the country and from foreign collectives [10]. Studies on the immunomodulatory potential of vitamin D in different MS phenotypes will clarify debated questions: specific group of MS patients eligible for vitamin D treatment; the effective and safe dose; and serum concentrations of the vitamin achieving suppression of the aberrant immune response.

## Materials and methods

Objective

To analyze the changes in the serum concentrations of 25-hydroxyvitamin D (25(OH)D), tumor necrosis factor-alpha (TNF-α), interferon-gamma (IFN-γ), interleukin 17 (IL17), transforming growth factor-beta (TGF-β), interleukin 4 (IL4), interleukin 10 (IL10) through the disease course, their relationship with the risk of developing MS and the severity of neurological deficit.

Design

The study is a case-control study. It is approved by the Ethics Commission at the Medical University Plovdiv, protocol No. 3 /07.05.2012/. The patients were selected as outpatients at the Center for Diagnosis and Treatment of MS at Saint George University Hospital - Plovdiv during the astronomical winter periods of October 2012 - May 2013, and October 2013 - May 2014. The voluntary participation of the persons is certified by written informed consent.

Control Group

The serum levels of 25(OH)D, TNF-α, IFN-γ, IL17, TGF-β, IL4, and IL10 were tested once during the described periods.

Patients

Double examination of the indicators during the relapse and remission (≥2 months of a consecutive attack). Patients with exacerbation are admitted to the Department of Neurology at Saint George University Hospital - Plovdiv. Methylprednisolone (Sopharma), 500 mg intravenous (IV) in the morning, in a course dose of 2500 mg, is administered as therapy.

Data

Data from 86 Caucasian individuals were analyzed. Of these, 40 controls (20 women, 20 men) and 46 patients (33 women, 13 men) were monitored inpatient at the time of the relapse and outpatient during remission.

Including criteria

Caucasians living between 41.5° north latitude and 25.17° east longitude, 18-50 years of age, patients with consecutive relapse and remission, with the Expanded Disability Status Scale (EDSS) rate 1.5-5.0.

Exclusion criteria

Primary and secondary progressive course, treatment with medications modifying the course of the disease in the previous year from the registration date, corticosteroids therapy 3 months before the first examination, treatment with vitamin D and preparations affecting its metabolism (oral contraceptives, hormone replacement, antiepileptic, laxatives, thiazide diuretics), accompanying autoimmune diseases, acute and chronic infections, liver, kidney, neoplastic, hyperparathyroidism, diabetes, hypercalcemia.

Control group

Clinically healthy individuals, comparable in age, who do not take contraceptives, hormones, laxatives, or multivitamins.

Methods

Clinical Method

Anamnestic data on the onset, duration of the disease and subsequent attacks, concomitant diseases, and received treatment. McDonald diagnostic criteria, severity of neurological deficit on the EDSS.

Definition of Relapse

Onset of new neurological symptoms or worsening of existing ones for ≥ 24 hours, deterioration of EDSS by ≥ 0.5 grade in the absence of infectious condition, and after 30 days of stable condition after a relapse.

Laboratory

Cytokines (ck) in pg/ml and 25(OH)D nmol/l serum concentrations were determined by immunosorbent assay with original enzyme-linked immunosorbent assay (ELISA) kits immunodiagnostic eBioscience-Austria, Germany. Each sample was tested with double analyses, read serially by ELISA-reader (Sirio-microplatereader, SEAC-Italy, λ-450 nm/reference λ=620 nm).

Statistics

The required number of observation units is determined by the formula (n_2=(t^2 σ_2^2)/((X_1 ) ®-(X_2 )-(t^2 σ_1^2)/n_1 ) ®\) with guarantee probability 0.95, α error 5%, β error 20%. The data is processed using Statistical Package for the Social Sciences (SPSS) 19.0. The quantitative measures are shown as mean and standard error (±SEM). The comparison of the results in the groups was carried out using the χ2 criterion - to establish a relationship between the studied factors, Independent Sample T-test, and Mann-Whitney at p<0.05 depending on the results of the Kolmogorov-Smirnov test. Regression statistical methods, multifactorial linear regression, and the Wilcoxon Signed Ranks test were used. The report from the Institute of Meteorology and Hydrology at the Bulgarian Academy of Sciences No. 2445/24.7.2015 shows that the data from the two periods are comparable: the number of clear and sunny days is in the zone of normal dispersion around the multi-year average value.

## Results

Forty-six patients with an average age of 37 ± 1.83 years and 40 healthy subjects with an average age of 31.67 ± 1.15 years were studied. The groups were comparable in age (t=2.38, p=0.02). The average age of the disease onset symptoms was 30.00 ± 1.29 years. The average duration of the disease was 7.17 ± 1.17, with a duration of MS up to 5 years 57%. The highest is the relative share of those registered until the end of the 3rd week of a consecutive attack (76.08; n=35). The mean EDSS score decreased reliably during remission compared to relapse (relapse 2.36 ± 0.11, remission 1.64 ± 0.41; t=5.95; p<0.0001). Of the healthy individuals, 57.5% have insufficient 25(OH)D ≥ 26 nmol/l, and 42.5% have a deficiency ≤ 25 nmol/l. OF them, 28.3% of the patients had insufficiency, 71.7% had 25(OH)D deficiency. The average concentrations of 25(OH)D in the patients and the healthy individuals are presented in Table 1.

**Table 1:** Average serum concentrations of 25(OH)D in patients in relapse, remission, and clinically healthy individuals. T1p: in comparison with the control; T1p1: comparison of the values in relapse and remission; 25(OH)D: 25-hydroxyvitamin D *statistical significance

Group	n	Mean ± Standard error of the mean (nmol/L)	Standard deviation	T	P	t_1_	P_1_
Control	40	37.39 ± 5.56	35.15	-	-	-	-
Patients in relapse	46	18.50 ± 2.42	16.38	2.60	0.009*	-	-
Patients in remission	46	27.05 ± 3.68	24.97	1.35	0.18	2.26	0.024*

A significantly low concentration of 25(OH)D was found in the patients in relapse when compared to the control group (t=2.60, p=0.009), there is a reliable increase of the indicator during the remission (t=2.26, p=0.024) compared to the relapse. A negative statistically significant relationship was registered between the mean levels of 25(OH)D and the deficiency severity during relapse (r= -0.416; p= 0.004). The assessment of the risk of developing MS depending on the exposure to the studied factor - serum concentration of 25(OH)D nmol/l is presented in Table 2.

**Table 2:** Serum levels of 25(OH)D nmol/l and risk of multiple sclerosis. 25(OH)D: 25-hydroxyvitamin D

Index	Healthy Control		Patients		Odds Ratio	95% CI	P
	number	%	number	%			
Serum levels of 25(ОН)D>26 nmol/l	23	57.5%	13	28.3%	1		
Serum levels of 25(ОН)D<=25nmol/l	17	42.5%	33	71.7%	3.43	(1.40;8.42)	0.006

In the studied cohort, vitamin D deficiency increases the overall risk 3.43 times. The comparison between the changes of cytokines in the patients during the two clinical phases with those in the healthy persons shows the following: significantly low levels of TNF-α, IL10, and IFN-γ in the patients in both phases; significantly higher TGF-b1 during remission compared to the control group as showed in Tables 3-4.

**Table 3:** Comparison between the levels of the pro-inflammatory cytokines during the two clinical phases with those of the controls.

Groups	#	Interleukin 17(pg/ml) Mean Rank	t	P	Tumor Necrosis Factor-Alpha (pg/ml) Mean Rank	t	P	Interferon-Gamma (pg/ml) Mean Rank	t	P
Control	40	41.28			50.44			54.13		
Patients in relapse	46	45.43	-0.77	>0.05	34.47	-2.40	<0.05	34.26	-3.68	<0.001
Patients in remission	46	46.91	-1.361	>0.05	37.25	-2.49	<0.05	30.84	-5.04	<0.001

**Table 4:** Comparison of anti-inflammatory cytokine levels during the two clinical phases with those of controls.

Groups	#	Interleukin 4 (pg/ml) Mean Rank	t	P	Interleukin 10 (pg/ml) Mean Rank	t	P	Transforming Growth Factor-Beta (pg/ml) Mean Rank	t	P
Control	40	44.48			53.73			39.15		
Patients in relapse	46	42.65	-0.33	>0.05	34.59	-3.55	<0.001	47.28	-1.50	>0.05
Patients in remission	46	46.15	-1.05	>0.05	36.02	-2.97	<0.01	48.76	-2.09	<0.05

Significantly increased concentrations of IL4 t=2.73, p=0.006, TGF-β t=2.74, p=0.009, were established during remission, with a tendency to increase IL10 compared to the levels in the relapse. During the period of clinical improvement, the mean levels of IFN-γ t=2.39, p=0.017 decreased statistically significantly and there is a weak trend for a decrease in IL17 and TNF-α compared to the concentration during relapse.

The analysis of causality data between the serum cytokine concentrations and 25(OH)D levels during the two phases shows a moderate strength, direct relationship between IL17 and 25(OH)D during the exacerbation (r=0.343; P = 0.02 ).

The evaluation of the relationship between cytokine changes and the severity of the neurological deficit at during relapse and remission shows a weak, statistically significant, direct relationship between the serum levels of TNF-α and the degree of disability during exacerbation r=0.301 t=0.042.

The relationship between the studied indicators and the deficit severity during relapse was evaluated by multivariate linear regression. Statistically significant, independent factors for the deficiency are 25(OH)D, TNF-α, and IL17 represented in Table 5.

**Table 5:** Multivariate regression model for the severity of neurological deficit during relapse. (F= 6.145, df=3, P=0.001) Unstandardized coefficients: Beta, standard error; standardized coefficients: Beta; 95.0% confidence Interval for B: lower/upper limit; dependent variable: EDSS relapse

Model		Beta	Standard Error	Beta	t	P	Lower Limit	Upper Limit
1	(Constant)	2.678	0.157		17.025	0.000	2.361	2.995
	25(OH)D relapse	-0.017	0.006	-0.376	-2.696	0.010	-0.030	-0.004
2	(Constant)	2.371	0.206		11.526	0.000	1.956	2.786
	25(OH)D relapse	-0.017	0.006	-0.371	-2.765	0.008	-0.029	-0.005
	TNFα relapse	0.086	0.039	0.293	2.190	0.034	0.007	0.166
3	(Constant)	2.476	0.203		12.176	0.000	2.066	2.887
	25(OH)D relapse	-0.012	0.006	-0.264	-1.913	0.063	-0.025	0.001
	TNFα relapse	0.109	0.039	0.371	2.776	0.008	0.030	0.188
	IL17 relapse	-0.005	0.002	-0.307	-2.160	0.037	-0.009	0.000

## Discussion

Mean serum 25(OH)D levels lower than 50 nmol/l are observed in 48-78% of all MS patients included in different MS populations [11]. This study shows a high frequency (71.7%) of individuals with 25(OH)D deficiency during the relapse phase. The mean levels of the vitamin were significantly decreased compared to controls and reliably increased during remission without reaching the values in the healthy subjects. The relationship between 25(OH)D deficiency during relapse and the EDSS scale was negative and significant and agrees with the thesis of a protective effect of the vitamin on the severity of a disability. A study in patients with primary-, secondary-progressive, and RR phenotypes reported a negative, significant relation between 25(OH)D levels and EDSS score [12]. A study in patients with an RR phenotype shows that low levels of 25(OH)D were associated with a higher grade of EDSS [8]. Fahmi et al. found reliably low concentrations of 25(OH)D in the patients compared to the healthy individuals and a more severe deficiency at low levels of the vitamin [13]. The risk assessment of developing MS shows a 3.43-fold increase in the indicator with 25(OH)D deficiency. Other authors in patients with 25(OH)D levels of 25 nmol/l reported a 50% reduction in risk when the concentration increased by 11 nmol/l [14]. A study among nurses reports a 40% reduction in the indicator in the group taking vitamin D [15]. Vitamin D supplementation is associated with a significantly lower risk of MS. The indicator is higher at vitamin levels 6-25 ng/ml and lower at levels 40-61 ng/ml [16]. During both periods, the patients had lower immune tolerance compared to the controls. During both phases, significantly lower levels of IFN-γ, IL10, IFN-γ, and significantly higher levels of TGF-β were reported in remission compared to the controls. Other observations report high values of TGF-β, IFN-γ, TNFα, and IL6 before exacerbation and compensatory over-activity is discussed [17-19]. During the two periods, significant differences were reported between the levels of certain cytokines: increase in IFN-γ during exacerbation and increase in IL4, TGF-β during remission. IFN-γ is a marker of Th1-driven pro-inflammatory activity. Different authors find higher secretion of IFN-γ from peripheral blood in patients in relapse, and higher serum levels compared to those in remission. Increased production of IFN-γ, an inducer of oligodendrocyte apoptosis, is detected in the brain lesions [20]. Significantly higher levels of IL4 and TGF-β during remission versus relapse are a manifestation of anti-inflammatory activity. Franciotta et al. in an identical comparison present a similar result for IL4 changes [21]. In our study, during the aggravated deficiency phase, the pro-inflammatory cytokines IFN-γ, and IL17 predominated and 71.7% of the patients were deficient in 25(OH)D. During the deficiency improvement phase, anti-inflammatory cytokines IL4, TGF-β dominate and 25(OH)D levels increase significantly. These results are grounds for discussing the immunomodulatory activity of 25(OH)D reported in the experimental models: 1.25(OH)2D directly shifts the Th1/Th2 balance to the dominance of Th2-mediated IL4 secretion [22]; high doses of 1.25(OH)2D are decisive for TGF-β production [23]. In patients with RR MS after therapy with 1.25(OH)2D there is inhibited synthesis of IFN-γ, TNF-α increased production of TGF-β, IL4 compared to untreated patients [24]. The analysis of the effect of the indicators on the deficit shows that 25(OH)D, TNF-α, and IL17 are significant independent factors for the EDSS severity during relapse. We found a trend for increased IL17 during relapse compared to remission. Other authors find a significantly higher relative proportion of Th17 lymphocytes during this period, higher serum and CSF levels in patients compared to controls, as well as insignificant differences in an identical comparison [25,26]. During both phases, TNF-α concentrations were without significant changes, unlike other data. Results are presented for increased levels of intracellular adhesion molecule 1 (ICAM1) and TNF-α receptors in the cerebrospinal fluid and serum of patients in relapse, activated production of TNF-α from peripheral blood prior to the relapse [27-30].

We believe that this study is the first to examine the relationship between alternations in the serum levels of the examined cytokines and vitamin D and the degree of neurological impairment in Bulgarian patients with RR MS. Given the proven vitamin D deficiency in our country`s general population and the findings on the immunomodulatory and protective effect of vitamin D in the analyzed patients, we believe this study is essential for identification of a specific group of MS patients suited for vitamin D treatment.

While we provide insights into the relationship between vitamin D, cytokines, and RR MS, our study was constrained by its single-center design, small patient population, and brief follow-up period and did not fully account for other plausible variables that might have affected the measured parameters such as genetic predisposition, nutrition, and lifestyle.

## Conclusions

25(OH)D has a protective effect on the risk of MS and the severity of neurological deficits. Serum concentrations of TNF-α and IL17 were significant and independent factors for the deficiency severity during relapse. Serum concentrations of IFN-g, TGF-b1, and IL4 are criteria for evaluating immune inflammatory activity during periods of relapse and remission.
